# CD56-Negative Aggressive NK Cell Leukemia Relapsing as Multiple Cranial Nerve Palsies: Case Report and Literature Review

**DOI:** 10.1155/2017/3724017

**Published:** 2017-10-15

**Authors:** M. Guerreiro, F. Príncipe, M. J. Teles, S. Fonseca, A. H. Santos, E. Fonseca, P. Gomes, C. Marques, M. Lima

**Affiliations:** ^1^Department of Clinical Hematology, Centro Hospitalar São João, Alameda Professor Hernâni Monteiro, 4200-319 Porto, Portugal; ^2^Department of Clinical Pathology, Centro Hospitalar São João, Alameda Professor Hernâni Monteiro, 4200-319 Porto, Portugal; ^3^Department of Hematology, Laboratory of Cytometry, Hospital de Santo António, Centro Hospitalar do Porto, Rua D. Manuel II, s/n, 4099-001 Porto, Portugal; ^4^Department of Pathology, Centro Hospitalar São João, Alameda Professor Hernâni Monteiro, 4200-319 Porto, Portugal

## Abstract

**Background:**

Aggressive natural killer cell leukemia (ANKL) is extremely rare and habitually manifests as a systemic disease with multiorgan failure that rapidly evolves to death. The neoplastic natural killer (NK) cells usually harbor the Epstein-Barr virus (EBV) with a latent viral infection pattern type II; they often have a cytoplasmic CD3*ε*^+^ and surface CD3^−^, CD2^+^, and CD56^+^ immunophenotype, and they show complex genetic abnormalities affecting multiple tumor suppressor genes and oncogenes. We present a rare case of CD56-negative ANKL and review the clinical and laboratorial criteria for the diagnosis, as well as the available therapies.

**Case Presentation:**

A European 36-year-old male presented with acute onset fever, pallor, weakness, and jaundice. He had hepatosplenomegaly, severe pancytopenia, hepatic cytolysis, and very high serum lactic dehydrogenase levels. The bone marrow studies resulted in the diagnosis of an EBV-positive, CD56-negative ANKL. The patient failed to respond to gemcitabine and cisplatin-based polychemotherapy, dying three months later with leukemic meningitis and multiple cranial nerves palsies.

**Conclusions:**

The diagnosis of ANKL is difficult and requires both clinical suspicion and an extensive laboratorial approach. Absence of CD56 expression on the neoplastic NK cells may impose difficulties in the diagnosis, which requires morphological, immunophenotypic, histopathological, immunohistochemical, cytogenetic, and molecular studies.

## 1. Background

Natural killer (NK) cell neoplasms are classified by the World Health Organization (WHO) into aggressive NK cell leukemia (ANKL) [1], extranodal NK/T cell lymphoma, nasal type (ENKTL) [2], and chronic lymphoproliferative disorders of NK cells [3], the latter being considered provisionally. ENKTL and ANKL are rare diseases, with higher prevalence in Asia and Central and South America. ENKTL usually present as a destructive tumor affecting the nose and upper aerodigestive tract or any organ or tissue in the body [4, 5]. ANKL is a very rare disease which comprises less than 0.1% of all lymphoid neoplasms [6] and usually manifests as a systemic disease with multiorgan involvement. The histopathological hallmark of these aggressive NK cell tumors is a polymorphic neoplastic infiltrate with angiocentricity, angiodestruction, and tissue necrosis. The tumor cells have cytoplasmic azurophilic granules containing cytotoxic molecules and they usually show a CD45^+^, CD2^+^, sCD3^−^, cytCD3*ε*^+^, CD56^+^, and CD16^−/+^ immunophenotype and Epstein-Barr virus- (EBV-) encoded membrane proteins; in addition, EBV-encoded small RNAs (EBERs) are usually detected on lymphoma cells by in situ hybridization, and complex chromosomal abnormalities are frequent. The rarity of the NK cell tumors limits our ability to standardize the procedures for the diagnosis and treatment and efforts should be made to encourage multi-institutional registries.

From the review of relevant literature, the patient reported herein is one of the rare cases of ANKL with a CD56-negative immunophenotype, the final event being meningeal infiltration with compromise of multiple cranial nerves; other clinical, laboratory, and biological features were those typically observed in this aggressive NK cell malignancy.

## 2. Case Presentation

### 2.1. Disease Presentation

**Figure 1:**
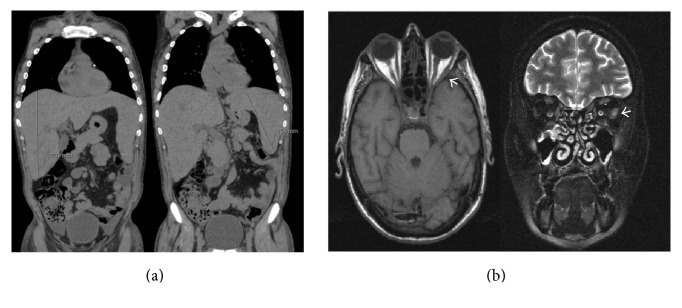
Thoracic-abdominal-pelvic computerized tomography scan showing the hepatosplenomegaly (liver and spleen longitudinal axis of 209 cm and 158 cm, resp.), without other significant abnormalities (a). Cerebral MR images showing swelling of the left lateral rectus muscle on a T2-weighted image, with no other additional findings (b).

A 36-year-old Caucasian Portuguese male was admitted to the hospital in October 2010, with fever (38°C), pallor, weakness, and jaundice. His medical history revealed chronic alcohol abuse. The physical examination showed hepatosplenomegaly and there were no palpable superficial lymph nodes. Blood counts demonstrated pancytopenia: white blood cells 1.80 × 10^9^/L, neutrophils 0.69 × 10^9^/L, lymphocytes 0.64 × 10^9^/L (no evidence for morphologically abnormal cells), platelets 46 × 10^9^/L, and hemoglobin 6.6 g/dl. Biochemistry analysis revealed markedly increased serum lactate dehydrogenase levels (LDH) 2815 IU/L (135–225 IU/L) and abnormal liver tests: total bilirubin (TB) 1.5 mg/dl (<1.2 mg/dL), aspartate transaminase (AST) 124 IU/L (10–37 IU/L), alanine transaminase (ALT) 62 IU/L (10–31 IU/L) and gama-glutamil transferase (GGT) 107 IU/L (10–49 IU/L); coagulation tests were normal. The abdominal computerized tomography (CT) scan confirmed the hepatosplenomegaly (liver and spleen longitudinal axis of 209 cm and 158 cm, resp.) and did not show other abnormalities (Figure 1(a)).

### 2.2. Laboratorial Investigation

**Figure 2:**
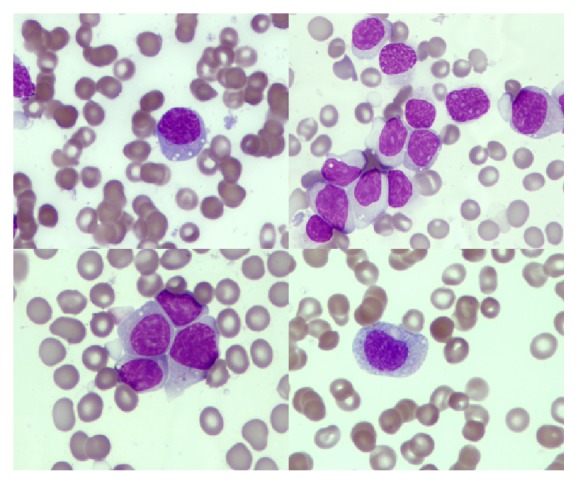
Bone marrow cytomorphology findings. Composite image of circulating leukemic cells obtained from Wright-Giemsa-stained peripheral blood smears.

The bone marrow (BM) aspirate had more than 80% of morphologically immature cells, with a pale or slightly basophilic cytoplasm sometimes with fine azurophilic granules and a nucleus with an immature chromatin, with one or two distinct nucleoli, and the first hypothesis for the diagnosis was that of an acute leukemia (Figure 2).

**Figure 3:**
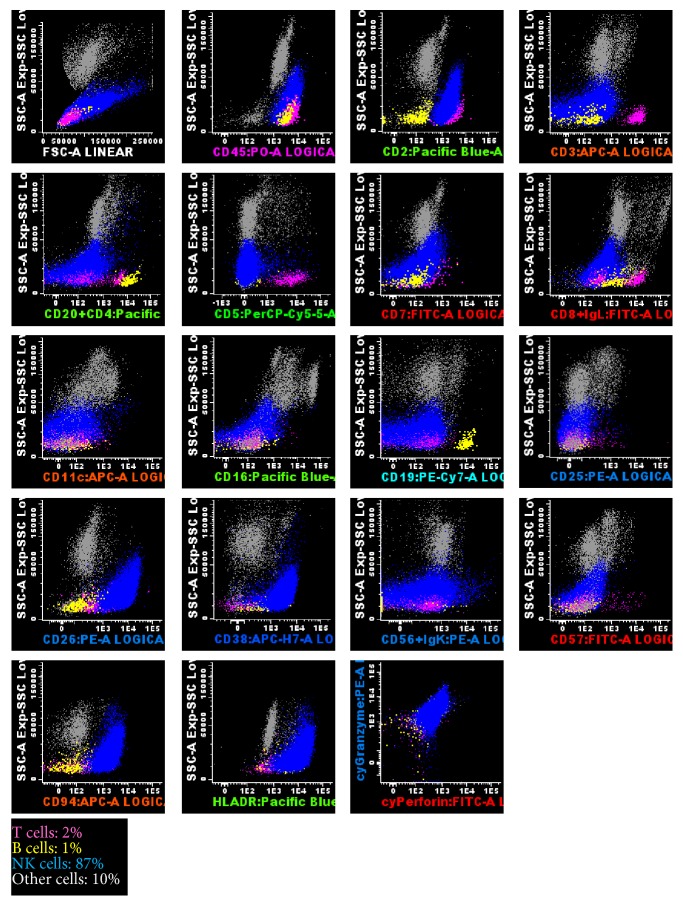
Flow cytometry studies in the bone marrow aspirate, using the EuroFlow lymphoid screening tube (LST) and antibody panel for NK cell chronic lymphoproliferative diseases (NK-CLPD), consisting of 8-color combinations of monoclonal antibodies (7), and a FACSCanto II flow cytometer (Becton Dickinson). Data analysis was performed using the Infinicyt software (Cytognos, Spain). Dot-plots illustrate the phenotypic features of the neoplastic NK cells. Blue dots correspond to the neoplastic NK cells whereas pink dots and yellow dots are the normal residual T cells and B cells present in the bone marrow sample. Other cells are represented as gray dots. The abnormal NK cells were positive for surface CD45 (high), CD2, CD26, CD38, CD94 (high), and HLA-DR and cytoplasmic granzyme B and perforin; and they were negative for surface CD3, CD4, CD5, CD7, CD8, CD11c, CD16, CD19, CD20, CD25, CD56, Ig kappa, and lambda chains.

Flow cytometry (FCM) analysis of the BM aspirate cells using the EuroFlow lymphoid screening tube (LST) and antibody panel for NK cell chronic lymphoproliferative diseases (NK-CLPD) [7], complemented with other cell surface markers, showed that the neoplastic cells were positive for CD45 (high), CD2, CD26, CD38, CD94 (high), and HLA-DR (high, heterogeneous) antigens, and negative for surface CD3, TCR, CD4, CD5, CD7, CD8, CD11b, CD11c, CD16, CD56, CD57, and CD161, as well as for cytoplasmic CD3; in addition, they express intracellular granzyme B and perforin (Figure 3). B cell (CD19, CD20, and CD79a), myeloid (CD13, CD14, CD15, CD64, CD65, and myeloperoxidase), immature (CD34, TdT), and dendritic (CD123) cell associated markers were all negative (data not shown). In the PB there were 10% of phenotypically abnormal CD45^+high^, CD2^+^, CD16^+low^, CD94^+high^, and HLA-DR^+^ NK cells, 19% of which expressed dimly and heterogeneously the CD56 molecule, at levels that were much lower than those observed in normal PB NK cells (data not shown). Flow cytometry propidium iodide based NK cell DNA studies revealed a diploid cell DNA content (DNA index 1.02) and a high proliferative rate (G0/G1 phases: 67.4%; S phase: 31.8%; G2/M phases: 0.8%). Polymerase Chain Reaction (PCR)* TCRG* gene rearrangements studies were consistent with a germ-line configuration.

**Figure 4:**
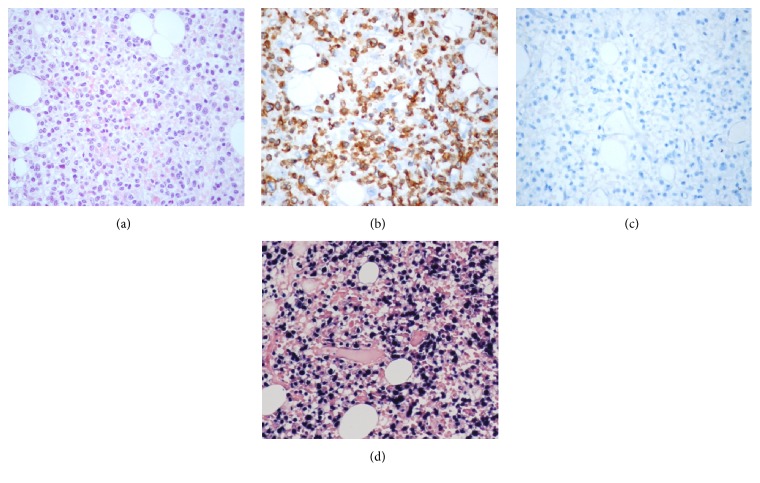
Bone marrow histological findings. Bone marrow biopsy section Wright-Giemsa stained (40x) (a). The lymphoid infiltrate is positive for CD3 epsilon (b) and negative for CD56 (c). In situ hybridization showing positivity of the neoplastic cells for EBER (d).

The BM biopsy revealed a hypercellular marrow infiltrated by CD45^+^, cytoplasmic CD3*ε*^+^, EBER^+^ and CD56, CD34, CD20, CD68, myeloperoxidase, and lysozyme-negative atypical lymphoid cells, interpreted individually given rise to the possibility of an immature EBV^+^ T cell leukemia (Figure 4).

**Figure 5:**
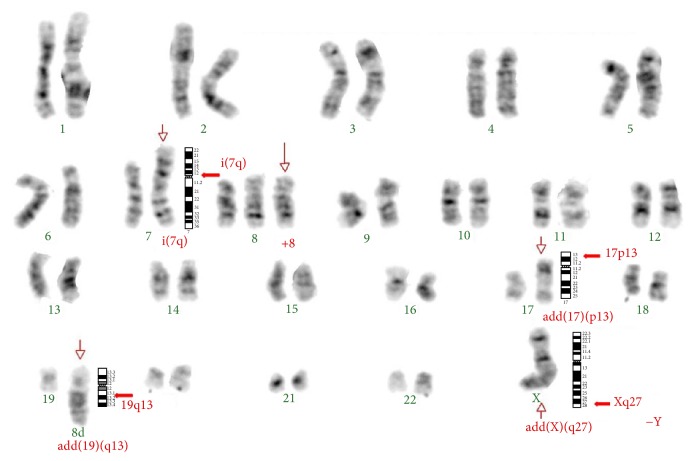
Bone marrow karyotype with chromosome banding analysis with complex aberrancies: 46,add(X)(q27),-Y, i(7)(q10),+8,add(17)(p13),add(19)(q13). Arrows indicate the chromosomal abnormalities.

The BM karyotype with chromosome banding analysis showed complex aberrancies: 46,add(X)(q27),-Y,i(7q), +8,add(17)(p13),add(19)(q13) (Figure 5).

Infection markers were negative for human immunodeficiency virus types 1 and 2, viral hepatitis B and C, and human T cell lymphotropic virus types 1 and 2. The whole blood EBV load, detected by quantitative PCR for viral DNA, was of 57 × 10^5^ copies/ml.

As no neurologic symptoms were present, central nervous system involvement was not evaluated.

### 2.3. Diagnosis

In view of these findings and according to the WHO classification of neoplasms of the hematopoietic and lymphoid tissues [1], the final diagnosis was an EBV^+^ ANKL, Ann Arbor stage IVB, high risk NKIPI (NK/T cell lymphoma international prognostic index), ECOG status 4.

### 2.4. Treatment and Disease Evolution

The patient was treated with a combination of gemcitabine (1 g/m^2^ days 1, 8, and 15), cisplatin (100 mg/m^2^ day 15), and methylprednisolone (1 g days 1–5) (GEM-P) repeated every 28 days [8].

The first BM reevaluation, performed on day 23 after the first chemotherapy course, revealed 0.5% of phenotypically abnormal (CD2^+^, CD56^−^, CD94^+^, and HLA-DR^+^) NK cells and a normal karyotype (46, XY). In addition, there was a marked decrease in the EBV load in the PB to 4 × 10^5^ (day 14) persisting at 6.6 × 10^5^ (day 21) copies/ml. Also, the BM aspirate showed 4.1 × 10^5^ EBV copies/ml. These changes correlated with an improvement in clinical status and blood analysis: WBC 5.14 × 10^9^/L, neutrophils 2.78 × 10^9^/L, lymphocytes 0.92 × 10^9^/L, platelets 45 × 10^9^/L, hemoglobin 8.9 g/dl (not dependent on blood or platelet transfusions), TB 1.10 mg/dl, AST 37 IU/L, ALT 39 IU/L, GGT 71 IU/L, and LDH 425 IU/L. A second evaluation of the BM aspirate after the second GEM-P showed only 0.09% of neoplastic NK cells. At that time, the BM biopsy provided evidence for a partial hematopoietic recovery, although intrasinusoidal niches of neoplastic NK cells were still observed; in addition, the BM karyotype was again abnormal, with different chromosomal aberrancies: 77,add(X)(q27),-Y,i(7)(q10),inc[5]/46,XY[15]. Similarly, an increase of the EBV load was observed (42 × 10^5^ copies/ml). Considering the refractoriness to GEM-P, it was decided to change the chemotherapy.

Nearly one month later, when the alternative schema was being discussed, the patient was admitted to urgency reporting loss of vision. At that time, the ophthalmologic and neurological examination revealed an almost total bilateral decline in visual acuity, markedly decreased pupil reflexes, right retinal detachment, and impaired right eye abduction compatible of palsy of the 6th right cranial nerve. Blood analyses were as follows: WBC 4.78 × 10^9^/L, neutrophils 2.14 × 10^9^/L, lymphocytes 1.69 × 10^9^/L, and platelets 155 × 10^9^/L, depending on regular blood transfusions, TB 0.88 mg/dl, AST 34 U/L, ALT 29 U/L, and LDH 429 IU/L. Abnormal NK cells had increased in blood (1.9% by FCM) and there was a marked increment on the EBV load (89 × 10^5^ copies/ml) and on the LDH (1587 IU/L). Head CT revealed a thickening of the right optic nerve, compatible with neoplastic infiltration, without evidence for other abnormalities in brain tissue and structures. Five days later he also developed dysphonia, dysphagia, impaired abduction of the left eye, and left eyelid ptosis and the head MRI showed a thickening of the left lateral rectus muscle (Figure 1(b)). At that time, there was clinical evidence of palsy of multiple cranial nerves (bilateral palsy of the 2nd and 8th left and right cranial pares, left palsy of the 3rd and 5th left cranial pares, and right palsy of the 6th and 7th right cranial pares). The CSF had 262 cells/*μ*l with 88% of atypical mononuclear cells, being consistent with leukemia meningitis. Immunophenotypic characterization was not performed, as on the day of the lumbar puncture flow cytometry was not available at our institution; EBV DNA analysis was not possible due to insufficient sample volume. At this point, no further chemotherapy was administered, and the strategy from here on was best supportive care. Death occurred a few days later, 107 days after the diagnosis.

## 3. Discussion and Conclusions

ANKL is an extremely rare and aggressive lymphoid neoplasm characterized by a proliferation of EBV-transformed mature NK cells, with a higher incidence in Asia and Central and South America [9]. A review of 73 cases of ANKL published in the English literature, from 1966 to 2003, reported a median age of 37 years at diagnosis and a slight male predominance, alongside acute onset of symptoms and a median survival of 61 days [10]. The largest series were published in 2004 [9] and 2013 [11], and they included only 22 and 43 patients, from Japan and China, respectively. To date, only around one hundred cases have been reported all over the world and the main disease manifestations are in concordance with the patient presented herein. Comparable clinical and pathological features were observed in 3 cases of EBV-negative ANKL that were recently reported [12].

Similarly to that typically found in other ANKL cases, our patient was very ill, with fever, cytopenias, liver function disturbances, and high levels of serum LDH; and, as in other ANKL cases, the disease affected mainly the BM and the PB, as well as the liver and the spleen. During the disease course, disseminated intravascular coagulation and hemophagocytic syndrome (HS) develop frequently, and multiorgan failure finally culminates with death. Unfortunately, the possibility of our patient having a HS was not considered at the diagnosis and, therefore, the criteria for diagnosis of HS were not fully evaluated [13]. Analyzing retrospectively, we verify that although some criteria for HS were present (splenomegaly, periods of fever ≥ 38.5°, and pancytopenia), some were not met (fibrinogen < 150 mg/dl and evidence of hemophagocytosis in the BM and liver) and others were not evaluated (ferritin, fasting triglycerides, NK cell activity, and soluble CD25). The available data applied to the HScore proposed by Fardet et al. corresponds to a probability of 30.1% of having HS [14]. In our patient, the final event was leukemic meningitis. Meningeal infiltration may be diffuse or focal, and it may manifest as signals/symptoms of increased intracranial pressure, visual disturbances, or cranial nerves paralysis. It may occur as part of the initial presentation of ANKL was previously documented in two published cases of ANKL with polycranial nerve palsies and peripheral neuropathy and meningitis [15, 16]; more commonly, however, meningeal spread occurs in the form of relapse, as in our patient.

The* immunophenotype* of ANKL cells is indistinguishable from that observed in ENKTL for the majority of the currently used markers, and previously published data would suggest that both diseases originate from CD56^+high^ and CD16^−/+low^ NK cells in most cases [17]. Lack of expression of CD56 observed in this case is uncharacteristic and may indicate a more immature NK cell immunophenotype, as more than 80% of the ANKL and ENKTL cases described to date were found to be CD56^+^; in addition, as in this case, most of the cases tested stained positively for surface CD2, as well as for cytoplasmic (but not surface) CD3*ε* and cytotoxic granule molecules, and about one-quarter of the ENKTL and half of ANKL cases are CD16^+^ [17].

A few cases of CD56-negative ENKTL have been reported in the literature [18–25] and case series have included some patients with CD56-negative ENKTL [26–28]. Li et al. found CD56 to be expressed in 90 of 118 (76.3%) patients with ENKTL and that the majority (83.3%) of patients with nasal ENKTL presented with CD56^+^ lymphoma, whereas patients with extranasal ENKTL of the upper aerodigestive tract were more likely to have CD56-negative tumors (53.6%) [26]. In a large cohort of 288 patients with early-stage ENKTL affecting the upper aerodigestive tract, from which 60 patients (20.8%) were categorized as CD56 negative, Wang et al. observed that CD56-negative ENKTL cases had significantly inferior survival outcomes [28]. In accordance, patients with CD56-negative ENKTL had significantly lower complete remission rates as compared to patients with CD56-positive ENKTL (60.8% versus 80.6%, resp.) and significantly lower 5-year and 10-year progression-free survival (PFS) and overall survival (OS) rates; furthermore, CD56 expression status proved to be an independent prognostic factor for PFS and had a trend to be independently correlated with OS [28].

To the best of our knowledge, so far, no individual cases of CD56 negative ANKL have been previously described in the literature, although a rare case of aggressive T cell large granular lymphocyte leukemia was recently reported [29], and a case series have revealed strong CD56 positivity in all but 5 cases of 43 (88.4%) of ANKL tested by flow cytometry (11). The only case of ANKL published in Portugal was not tested for CD56 expression [30]. Absence of CD56 expression imposes diagnostic difficulties, as the phenotypic criteria routinely used to identify NK cells usually rely on the positivity for CD56. In addition, NK cell neoplasms are sometimes erroneously classified as T cell neoplasms in BM and tissue biopsies, as NK cells frequently have epsilon chains of CD3 in the cytoplasm and, therefore, they may stain positively for CD3 in immunohistochemistry of paraffin sections [31].

The neoplastic NK cells are almost invariably* latently infected with the EBV*, thereby expressing the EBER-1 and EBER-2, the EBV nuclear antigen 1, and, usually, the latent membrane proteins 1 and 2, detected by in situ hybridization or immunohistochemistry on tissue biopsies, respectively. As in the case reported herein, high levels of EBV DNA copies are usually found in the blood [11], and the viral load can be followed up over time using quantitative PCR assays, as previously described for posttransplant lymphoproliferative disorders [32]. Overall, the EBV infection was documented in more than 85% of ANKL cases.

Currently,* genetic abnormalities *specific for ENKTL and ANKL have not yet been identified, although complex chromosomal aberrancies occur in a large fraction of cases [33]. Loss of heterozygosity at chromosomes 6q, 13q, 11q, and 17p is frequent [34], with a recurrent deletion on 6q in the target area 6q21–25 [35]. Consequently, multiple genes are affected, often due to gene deletion, mutation, or methylation, which include tumor suppressor genes and oncogenes [36]. An array-based comparative genomic hybridization study indicates that the genetic aberrancies recurrently observed in ANKL, which include loss of 7p15.1–p22.3 and 17p13.1 and gain of 1q, are different from those found in ENKTL [37]. Among the complex karyotype abnormalities found in our patient, the gain of genetic material on chromosomes 17p13 and 19q13 and the isochromosome 7q might affect genes known to be involved in a wide variety of human cancers. For instance, genes located at 17p13.1 include those codifying for the aurora kinase B (AURKB), a centrosome-associated kinase that plays an important role as regulator of chromosome segregation and cytokinesis, and, for the tumor protein p53, a protein that causes cells with damaged DNA to arrest at the G1 phase of cell cycle (TP53). Deletions on* TP53*, a tumor suppressor gene, are frequently found in neoplastic NK cells and have been associated with more advanced disease, suggesting a secondary oncogenic event rather than a triggering mechanism [38]. In addition, genes located at the 19q13 are the* BAX* and* BCL3* apoptosis related genes [39], genes involved in cell cycle progression that codify for cyclins, such as* CCNE1*, and genes codifying for transcription factors, such as* FOSB* [40], among others [41]. Finally, the isochromosome 7q, the primary cytogenetic abnormality in hepatosplenic gamma/delta T cell lymphoma, where the* TCRB* gene located at 7q35 is affected, was also previously described in 2 cases of ENKTL [42].

Although some treatment algorithms have been proposed for ENKTL and ANKL [43], there is no curative and uniformly accepted approach [44, 45]. Standard anthracycline-based schemas such as CHOP (cyclophosphamide, doxorubicin, vincristine, and prednisone) are known to be ineffective, due to the expression of proteins associated with multidrug resistance [46, 47]. L-Asparaginase, etoposide, and methotrexate based schemas, such as the SMILE regimen (steroids, methotrexate, ifosfamide, L-asparaginase, and etoposide), have shown promising results [48, 49], and a phase II study for evaluating the effectiveness of the association of L-asparaginase, methotrexate, and dexamethasone for relapsing and/or refractory ENKTL has recently been done [50].

This patient was refractory to GEM-P, a gemcitabine and cisplatin-based schema that was previously tested in patients with peripheral T cell lymphoma, with encouraging results and acceptable toxicity [8]. An observational retrospective study on the use of gemcitabine in pretreated/refractory ENKTL has been recently completed, but the results are not yet available so far [51]; in addition two randomized controlled multicenter clinical trials are now evaluating the efficacy of the DDGP (gemcitabine, pegaspargase, cisplatin, and dexamethasone) regimen for patients with ENKTL [52, 53]. The role of stem cell transplantation in the treatment of the NK cell neoplasms has been studied retrospectively by the NK cell Tumor Study Group; results have indicated a possible beneficial effect, but the criteria for transplantation were not uniformly accepted [54]. Investigations on future therapies are under progress and possible targets include survivin [55, 56] and the AURKA (Aurora Kinase A) and NOTCH-1 (Notch Homologue 1) oncogenic pathways [57]; modulation of gene expression with hypomethylating agents [58], immunoconjugates of anti-CD56 monoclonal antibodies [59], and inhibitors of tubule polymerization [42] are other possibilities.

In conclusion, the rarity of the ANKL and its clinical aggressiveness limit our ability to standardize the procedures for the diagnosis and clinical management and efforts should be made to encourage multi-institutional registries and clinical trials. CD56-negative cases impose diagnostic difficulties, being correctly diagnosed only if the immunophenotype of the neoplastic cells is exhaustively studied.

## References

[1] Chan J., Jan E., Ralfkiaer E., Swerdlow S., Campo E., Harris N. (2008). Aggressive NK-cell leukaemia. *World Health Organization Classification of Tumours of Haematopoietic and Lymphoid Tissues*.

[2] Chan J., Quintanilla-Martinez L., Ferry J., Swerdlow S., Campo E., Harris N. (2008). Extranodal NK/T-cell lymphoma, nasal type. *World Health Organization Classification of Tumours of Haematopoietic and Lymphoid Tissues*.

[3] Villamor N., Chan L., Foucar K., Swerdlow S., Campo E., Harris N. (2008). Chronic lymphoproliferative disorders of NK cells. *World Health Organization Classification of Tumours of Haematopoietic and Lymphoid Tissues*.

[4] Lima M., Gonçalves C., Dos Anjos Teixeira M. (2001). Aggressive natural-killer cell lymphoma presenting with skin lesions, breast nodule, suprarenal masses and life-threatening pericardial and pleural effusions. *Leukemia and Lymphoma*.

[5] Lima M., Spínola A., Fonseca S. (2015). Aggressive mature natural killer cell neoplasms: report on a series of 12 European patients with emphasis on flow cytometry based immunophenotype and DNA content of neoplastic natural killer cells. *Leukemia & Lymphoma*.

[6] Chan J. K. C., Jane E. S., Ralfkiaer E., Ko Y. H., Swerdlow S., Campo E., Harris N. (2008). Aggressive NK-cell leukaemia. *World Health Organization Classification of Tumours of Haematopoieticand Lymphoid Tissues*.

[7] Van Dongen J. J. M., Lhermitte L., Böttcher S. (2012). EuroFlow antibody panels for standardized n-dimensional flow cytometric immunophenotyping of normal, reactive and malignant leukocytes. *Leukemia*.

[8] Arkenau H.-T., Chong G., Cunningham D. (2007). Gemcitabine, cisplatin and methylprednisolone for the treatment of patients with peripheral T-cell lymphoma: the Royal Marsden Hospital experience. *Haematologica*.

[9] Suzuki R., Suzumiya J., Nakamura S. (2004). Aggressive natural killer-cell leukemia revisited: Large granular lymphocyte leukemia of cytotoxic NK cells. *Leukemia*.

[10] Ruskova A. K., Thula R., Chan G. T. C. (2004). Aggressive natural killer-cell leukemia: Report of five cases and review of the literature. *Leukemia and Lymphoma*.

[11] Jiang N.-G., Jin Y.-M., Niu Q., Zeng T.-T., Su J., Zhu H.-L. (2013). Flow cytometric immunophenotyping is of great value to diagnosis of natural killer cell neoplasms involving bone marrow and peripheral blood. *Annals of Hematology*.

[12] Gao J., Behdad A., Ji P., Wolniak K. L., Frankfurt O., Chen Y. (2017). EBV-negative aggressive NK-cell leukemia/lymphoma: a clinical and pathological study from a single institution. *Modern Pathology*.

[13] Henter J.-I., Horne A., Aric M., Aricó M. (2007). HLH-2004: diagnostic and therapeutic guidelines for hemophagocytic lymphohistiocytosis. *Pediatric Blood Cancer*.

[14] Fardet L., Galicier L., Lambotte O. (2014). Development and validation of the HScore, a score for the diagnosis of reactive hemophagocytic syndrome. *Arthritis & Rheumatology*.

[15] Taniguchi S., Machi M., Ohno Y. (1996). Epstein-Barr virus-associated CD3- large granular lymphocyte leukemia presenting with polycranial nerve palsies [5]. *Blood*.

[16] Lackowski D., Koberda J. L., DeLoughery T. G., So Y. (1998). Natural killer cell leukemia as a cause of peripheral neuropathy and meningitis: Case report. *Neurology*.

[17] Lima M. (2015). Extranodal NK/T cell lymphoma and aggressive NK cell leukaemia: Evidence for their origin on CD56+bright CD16-/+dim NK cells. *Pathology*.

[18] Chang B. H., Stork L., Fan G. (2008). A unique case of adolescent CD56-negative extranodal NK/T-cell lymphoma, nasal type. *Pediatric and Developmental Pathology*.

[19] Chang B., Stork L., Fan G. (2006). A unique case of Adolescent CD56-negative nasal extranodal NK/T-cell Lymphoma. *Pediatric and Developmental Pathology*.

[20] Katsaounis P., Alexopoulou A., Dourakis S. P. (2008). An extranodal NK/T cell lymphoma, nasal type, with specific immunophenotypic and genotypic features. *International Journal of Hematology*.

[21] Lan M. X., Zhen Z. X., Ming W. H. (2009). CD56-negative extranodal nasal type of natural killerT-cell lymphoma with extranasal skin involvement. *Leukemia and Lymphoma*.

[22] Miles R. R., Afify Z., Yaish H. (2010). CD56-negative extranodal nasal type NK/T-cell lymphoma. *Pediatric Blood Cancer*.

[23] Pine R. R., Clark J. D., Sokol J. A. (2013). CD56 negative extranodal NK/T-cell lymphoma of the orbit mimicking orbital cellulitis. *Orbit*.

[24] Baek Y.-S., Shin S.-H., Yi H.-G. (2014). Cardiac involvement in CD56 negative primary pancreatic extranodal NK/T-cell lymphoma, nasal type, presenting with ventricular tachycardia during the early stages of chemotherapy. *Internal Medicine*.

[25] Kim H. J., Kim S. H., Oh S. H. (2015). CD56-negative extranodal NK/T-cell lymphoma, nasal type, with extranasal cutaneous involvement. *Annals of Dermatology*.

[26] Li Y.-X., Wang H., Feng X.-L. (2011). Immunophenotypic characteristics and clinical relevance of CD56+ and CD56- extranodal nasal-type natural killer/T-cell lymphoma. *Leukemia and Lymphoma*.

[27] Pongpruttipan T., Kummalue T., Bedavanija A. (2011). Aberrant antigenic expression in extranodal NK/T-cell lymphoma: A multi-parameter study from Thailand. *Diagnostic Pathology*.

[28] Wang L., Wang Z., Xia Z.-J., Lu Y., Huang H.-Q., Zhang Y.-J. (2015). CD56-negative extranodal NK/T cell lymphoma should be regarded as a distinct subtype with poor prognosis. *Tumor Biology*.

[29] Sylvia M. T., Jacob S. E., Basu D. (2016). CD56 Negative Aggressive T Cell Large Granular Lymphocytic Leukemia. *Indian Journal of Hematology Blood Transfusion*.

[30] Sousa J., Cabezuelo L., Almeida S. (2011). Aggressive NK/T cell leukemia/lymphoma associated with EBV. *Acta Médica Portuguesa*.

[31] Gaal K., Sun N. C. J., Hernandez A. M., Arber D. A. (2000). Sinonasal NK/T-cell lymphomas in the United States. *American Journal of Surgical Pathology*.

[32] Gulley M. L., Tang W. (2010). Using epstein-barr viral load assays to diagnose, monitor, and prevent posttransplant lymphoproliferative disorder. *Clinical Microbiology Reviews*.

[33] Liang X., Graham D. K. (2008). Natural killer cell neoplasms. *Cancer*.

[34] Siu L. L., Chan V., Chan J. K., Wong K., Liang R., Kwong Y. (2000). Consistent Patterns of Allelic Loss in Natural Killer Cell Lymphoma. *The American Journal of Pathology*.

[35] Sun H. S., Su I.-J., Lin Y.-C., Chen J.-S., Fang S.-Y. (2003). A 2.6 Mb interval on chromosome 6q25.2-q25.3 is commonly deleted in human nasal natural killer/T-cell lymphoma. *British Journal of Haematology*.

[36] Huang Y., de Reyniès A., de Leval L. (2010). Gene expression profiling identifies emerging oncogenic pathways operating in extranodal NK/T-cell lymphoma, nasal type. *Blood*.

[37] Nakashima Y., Tagawa H., Suzuki R. (2005). Genome-wide array-based comparative genomic hybridization of natural killer cell lymphoma/leukemia: different genomic alteration patterns of aggressive NK-cell leukemia and extranodal NK/T-cell lymphoma, nasal type. *Genes Chromosomes and Cancer*.

[38] Quintanilla-Martinez L., Kremer M., Keller G. (2001). p53 mutations in nasal natural killer/t-cell lymphoma from mexico: Association with large cell morphology and advanced disease. *American Journal of Pathology*.

[39] Llambi F., Green D. R. (2011). Apoptosis and oncogenesis: give and take in the BCL-2 family. *Current Opinion in Genetics Development*.

[40] Eferl R., Wagner E. F. (2003). AP-1: a double-edged sword in tumorigenesis. *Nature Reviews Cancer*.

[41] Cancer Genetics Web.

[42] Feldman A. L., Law M., Grogg K. L. (2008). Incidence of TCR and TCL1 gene translocations and isochromosome 7q in peripheral T-cell lymphomas using fluorescence in situ hybridization. *American Journal of Clinical Pathology*.

[43] Tse E., Kwong Y.-L. (2011). Treatment algorithms for mature T-cell and natural killer-cell neoplasms. *Future Oncology*.

[44] Oshimi K. (2007). Progress in understanding and managing natural killer-cell malignancies. *British Journal of Haematology*.

[45] Suzuki R. (2010). Treatment of advanced extranodal NK/T cell lymphoma, nasal-type and aggressive NK-cell leukemia. *International Journal of Hematology*.

[46] Saglam A., Hayran M., Uner A. H. (2008). Immunohistochemical expression of multidrug resistance proteins in mature T/NK-cell lymphomas. *APMIS*.

[47] Huang W.-T., Huang C.-C., Weng S.-W., Eng H.-L. (2009). Expression of the multidrug resistance protein MRP and the lung-resistance protein LRP in nasal NK/T cell lymphoma: Further exploring the role of P53 and WT1 gene. *Pathology*.

[48] Yamaguchi M., Kwong Y.-L., Kim W. S. (2011). Phase II study of SMILE chemotherapy for newly diagnosed stage IV, relapsed, or refractory extranodal Natural Killer (NK)/T-cell lymphoma, nasal type: The NK-cell tumor study group study. *Journal of Clinical Oncology*.

[49] Kwong Y.-L., Kim W. S., Lim S. T. (2012). SMILE for natural killer/T-cell lymphoma: analysis of safety and efficacy from the Asia Lymphoma Study Group. *Blood*.

[50] Association of L-asparaginase-Methotrexate-Dexamethasone for Nasal and Nasal-type Natural Killer (NK)-T-cell Lymphoma. NCT00283985.

[51] Gemcitabine in NK/T Cell Lymphoma. NCT01660568.

[52] Treatment of Natural Killer/T Cell Lymphoma-I/II (CTTNKTL-I/II). NCT01501136.

[53] Treatment of Natural Killer/T Cell Lymphoma-III/IV (CTTNKTL-III/IV). NCT01501149.

[54] Suzuki R., Suzumiya J., Nakamura S. (2006). Hematopoietic stem cell transplantation for natural killer-cell lineage neoplasms. *Bone Marrow Transplantation*.

[55] Liu X., Ryland L., Yang J. (2010). Targeting of survivin by nanoliposomal ceramide induces complete remission in a rat model of NK-LGL leukemia. *Blood*.

[56] Ng S.-B., Selvarajan V., Huang G. (2011). Activated oncogenic pathways and therapeutic targets in extranodal nasal-type NK/T cell lymphoma revealed by gene expression profiling. *Journal of Pathology*.

[57] Iqbal J., Weisenburger D. D., Chowdhury A. (2010). Natural killer cell lymphoma shares strikingly similar molecular features with a group of non-hepatosplenic *γδ* T-cell lymphoma and is highly sensitive to a novel aurora kinase A inhibitor in vitro. *Leukemia*.

[58] Schmiedel B. J., Arélin V., Gruenebach F., Krusch M., Schmidt S. M., Salih H. R. (2011). Azacytidine impairs NK cell reactivity while decitabine augments NK cell responsiveness toward stimulation. *International Journal of Cancer*.

[59] Ishitsuka K., Jimi S., Goldmacher V. S., Ab O., Tamura K. (2008). Targeting CD56 by the maytansinoid immunoconjugate IMGN901 (huN901-DM1): A potential therapeutic modality implication against natural killer/T cell malignancy. *British Journal of Haematology*.

